# Unobtrusive Health Monitoring in Private Spaces: The Smart Home

**DOI:** 10.3390/s21030864

**Published:** 2021-01-28

**Authors:** Ju Wang, Nicolai Spicher, Joana M. Warnecke, Mostafa Haghi, Jonas Schwartze, Thomas M. Deserno

**Affiliations:** 1 Peter L. Reichertz Institute for Medical Informatics of TU Braunschweig and Hannover Medical School, Muehlenpfordtstr. 23, D-38106 Braunschweig, Lower Saxony, Germany; nicolai.spicher@plri.de (N.S.); joana.warnecke@plri.de (J.M.W.); mostafa.haghi@plri.de (M.H.); jonas.schwartze@plri.de (J.S.); thomas.deserno@plri.de (T.M.D.); 2 Wohnungsentwicklung und Forschung, Nibelungen-Wohnbau-GmbH, Freyastr. 10, D-38106 Braunschweig, Lower Saxony, Germany

**Keywords:** sensor, smart home, health monitoring, elderly, patient, ambient assisted living

## Abstract

With the advances in sensor technology, big data, and artificial intelligence, unobtrusive in-home health monitoring has been a research focus for decades. Following up our research on smart vehicles, within the framework of unobtrusive health monitoring in private spaces, this work attempts to provide a guide to current sensor technology for unobtrusive in-home monitoring by a literature review of the state of the art and to answer, in particular, the questions: (1) What types of sensors can be used for unobtrusive in-home health data acquisition? (2) Where should the sensors be placed? (3) What data can be monitored in a smart home? (4) How can the obtained data support the monitoring functions? We conducted a retrospective literature review and summarized the state-of-the-art research on leveraging sensor technology for unobtrusive in-home health monitoring. For structured analysis, we developed a four-category terminology (location, unobtrusive sensor, data, and monitoring functions). We acquired 912 unique articles from four relevant databases (ACM Digital Lib, IEEE Xplore, PubMed, and Scopus) and screened them for relevance, resulting in n=55 papers analyzed in a structured manner using the terminology. The results delivered 25 types of sensors (motion sensor, contact sensor, pressure sensor, electrical current sensor, etc.) that can be deployed within rooms, static facilities, or electric appliances in an ambient way. While behavioral data (e.g., presence (n=38), time spent on activities (n=18)) can be acquired effortlessly, physiological parameters (e.g., heart rate, respiratory rate) are measurable on a limited scale (n=5). Behavioral data contribute to functional monitoring. Emergency monitoring can be built up on behavioral and environmental data. Acquired physiological parameters allow reasonable monitoring of physiological functions to a limited extent. Environmental data and behavioral data also detect safety and security abnormalities. Social interaction monitoring relies mainly on direct monitoring of tools of communication (smartphone; computer). In summary, convincing proof of a clear effect of these monitoring functions on clinical outcome with a large sample size and long-term monitoring is still lacking.

## 1. Introduction

Living environments with limited public access, such as a home or a privately-owned car, form private spaces where people spend much time on daily activities [1]. In many cases, private spaces imply far more than ordinary living. For patients with chronic diseases or patients post-operationally discharged, their homes are places for not only living but also rehabilitation, and—in the future—could also facilitate medical diagnosis and therapy [2,3,4]. Health monitoring in private spaces will benefit the subjects who require assistance, such as the elderly, patients, and disabled persons.

The Internet of things (IoT) and ambient assisted living (AAL) enable the sensing of many aspects of our life, particularly including health-relevant information [5]. A sensor-enhanced private space can provide unobtrusive health monitoring. We defined unobtrusive health monitoring as ambient using sensor technology to collect human health-related data without introducing any inconveniences to everyday life [6]. Given this definition, wearable devices are not considered unobtrusive, as the adherence to wearing them introduces an additional burden to the users. Even in the case of a user-friendly integration of the sensors into a smart watch or wristband, they still need to be recharged or configured. Within the framework of unobtrusive health monitoring in private spaces, our previous work investigated the up-to-date research on leveraging sensor technology in smart vehicles [7]. In this work, we extend this thematic series in the framework of unobtrusive health monitoring by focusing on the sensor-enhanced private spaces, namely, smart homes.

A pivotal feature of unobtrusive health monitoring is continuity. Conventional approaches collect health information (e.g., morphological and functional performance assessments) at the point-of-care over time intervals ranging from months to years. In contrast, a smart home can continuously or over shorter time intervals monitor its resident’s health status while the resident is doing daily activities, unaffected by the measurements. This monitoring may capture a comprehensive picture of a person’s health and functional status and critical changes or events [8]. As is known, the activities of daily living (ADLs) reflect the behavioral routines. However, a human may not be able to pay close attention to how well an individual performs the ADLs and therefore spot subtle changes that may signal a pattern of decline [9]. Continuous health monitoring could ensure that subtle changes are not overlooked. Besides, studies have shown that the ambient in-home health monitoring technologies are feasible and well-accepted [10,11].

Smart home research and relevant topics have been reviewed from different perspectives in the past. Demiris et al. categorized health-related smart home technologies into physiological monitoring, functional monitoring or emergency detection and response, safety monitoring and assistance, security monitoring and assistance, social interaction monitoring and assistance, and cognitive and sensory assistance [12]. Majumder et al. analyzed the smart home monitoring technologies for the elderly and summarized the monitoring of resident activity, the home environment, resident health, and home appliances [13]. Liu et al. assessed the level of evidence in using smart home technology to support different health concerns, such as ADL monitoring, chronic obstructive pulmonary disease (COPD), cognitive decline and mental health struggles, fall prevention, and monitoring heart conditions; and concluded that the technology readiness for smart home and health monitoring is still low [14]. Cedillo et al. explored the relation between health concerns and different AAL technology [15]. Rashidi et al. summarized the AAL tools (smart homes, wearable sensors, and robotics) for older adults [16]. Recently, Rodrigues et al. addressed the requirements for the development of smart healthcare environments for physiological and behavior monitoring [17]. Stavropoulos et al. examined IoT wearable sensors and some smart home devices in elderly care, and categorized the sensing approaches according to healthcare aspects, ranging from specific ailments to general eldercare [18]. In addition, the authors proposed a case study classification taxonomy, which can be a reference for similar work.

The concept of unobtrusive health monitoring aims at measuring health parameters without interfering with the subject. However, this concept is not on the focus of existing reviews. A review of the current sensor technology for unobtrusive in-home monitoring is yet in demand. Therefore, this literature review aims to provide a snapshot of the state-of-the-art sensor technology in unobtrusive health monitoring. We focus on the subject groups of the elderly, patients, and disabled individuals, who need to be paid close attention in their everyday lives. In particular, the following questions will be answered: (1) What types of sensors can be used for unobtrusive in-home health data acquisition? (2) Where should the sensors be placed? (3) Which data can be monitored in the smart home? (4) How can the obtained data support the monitoring functions?

## 2. Methods

### 2.1. Terminology of Unobtrusive In-Home Health Monitoring

The term ADLs is defined as the self-care activities that are necessary for health maintenance and independent living [19]. Basic and instrumental ADLs (BADLs and IADLs) are the typical classes. The BADLs refer to life-sustaining self-care activities (e.g., feeding, grooming, bathing, dressing, toileting, and ambulation); the IADLs are more complex activities necessary for independent living (e.g., using telephones, preparing meals, shopping, managing finances, taking medications, and driving) [19]. ADLs indicate an individual’s health status, as the performance of ADLs depends on cognitive (e.g., reasoning and planning), motor (e.g., balance and dexterity), and perceptual abilities (e.g., hearing and seeing) [20].

**Figure 1:**
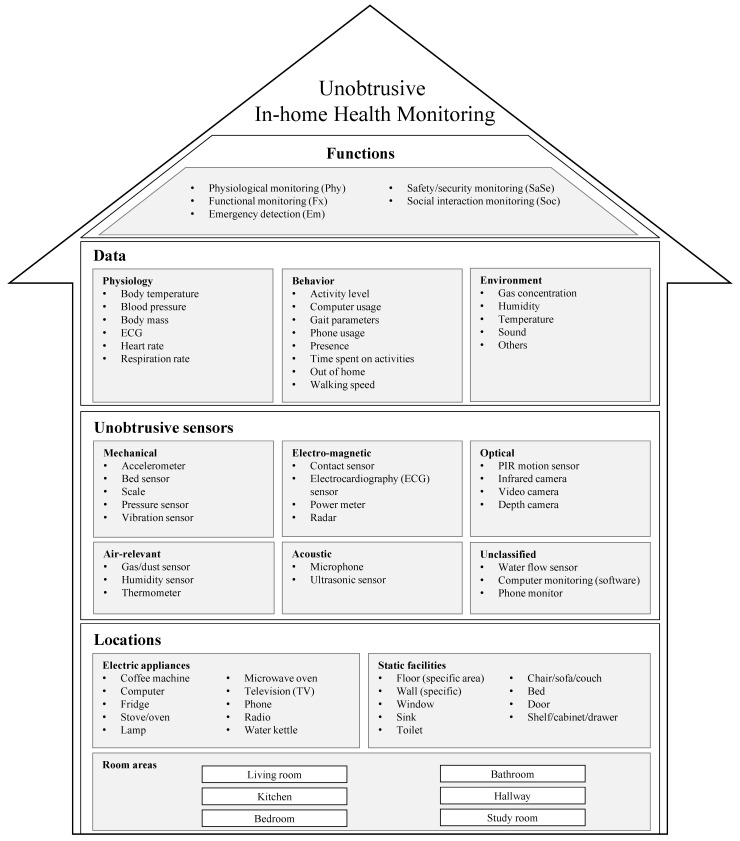
Terminology of unobtrusive in-home health monitoring.

Conventionally, we assess the ability to perform ADLs with clinical instruments, such as the Barthel index or the Lawton IADL scale [21,22]. Interactions with many in-home objects (e.g., toilet, oven, bed, or telephone) are unavoidable while performing ADLs. Thereby, we believe that sensors attached/integrated to these objects can enable automatic assessments of ADLs to a certain extent. To comprehensively understand different approaches, we have to consider the monitoring context, including sensor placement and data sources [23]. Accordingly, we propose a terminology that covers unobtrusive sensors, their locations, the data that can be obtained, and the potential monitoring functions (Figure 1). Hereby the term unobtrusive is regarded as unnoticed—i.e., the acquiring of health data is accomplished while the monitored individual is doing everyday activities as usual. For instance, heart rate can be measured without notice when a person is watching TV while sitting on a sofa integrating capacitive electrocardiography (ECG) electrodes.

- Location refers to the objects where the sensors can be unobtrusively deployed or integrated. It can be broken down into:
  - Room areas, which denotes rooms and other large spaces such as a hallway;
  - Static facilities, which consist of objects with fixed locations but usually without any electrical supply—e.g., furniture, windows, toilets, and sink;
  - Electric appliances have electricity, but may be fixed (e.g, oven) or unfixed (e.g., phone).
- Unobtrusive sensors refer to the sensing devices that can be unobtrusively deployed in the locations introduced above, consisting of mechanical, electro-magnetic, optical, acoustic, and air sensors [13].
- Data are the outputs directly from the sensors or derived values from data processing or analysis. Physiological, behavioral, and environmental data can be acquired from the sensors.
- Functions refer to the possible services that can be delivered through the monitoring system. We formalized the functions based on Demiris’ definitions [12].
  - Physiological monitoring (Phy) refers to the data collection and analysis of physiological measurements (e.g., heart rate, respiration rate, and body temperature).
  - Functional monitoring (Fx) refers to the data collection and analysis on functional measurements of BADLs and IADLs (e.g., activity level, motion, gait, and meal intake).
  - Emergency detection (Em) refers to detecting abnormal or critical situations that need immediate intervention (e.g., falls).
  - Safety and security detection (SaSe) refers to the detection of environmental hazards (e.g., fire and gas leak) and human threats (e.g., intruders).
  - Social interaction monitoring (Soc) refers to social interactions (e.g., phone calls, visitors, and social activities).

### 2.2. Literature Retrieval

The search string we developed reflects two aspects (Appendix A):

**Unobtrusive monitoring technologies** consist of terms on the technologies applied for unobtrusive sensor monitoring, such as “smart home”; “in-home monitoring”; “home-based monitoring”; “continuous assessment”; “ambient assisted living”; “intelligent monitoring.”

**People that are monitored** consist of the terms on the target groups who need healthcare support in their everyday life, such as “patient”; “disability”; “disabled”; “elderly”; “older people.”

We constructed the search term by connecting the terms within and across each aspect with logic operators OR and AND, respectively. We applied the search string to four databases, i.e., ACM Digital Lib, IEEE Xplore, PubMed, and Scopus. To reflect only up-to-date research, we defined the publishing date span as the last decade (May 2010–April 2020). We restricted the query to results written in English. Subsequently, we combined all returned records, removed duplicates, and screened the titles and abstracts according to the Review Criteria and excluded irrelevant records. Afterward, we analyzed the full texts with the Terminology of Unobtrusive In-Home Health Monitoring.

### 2.3. Review Criteria

As several persons performed a two-stage review, we defined the following criteria to maintain consistency:

- Inclusion
  - Unobtrusive sensors were part of the method;
  - The sensors were used to collect either behavioral, physiological, or environmental health-relevant data;
  - The monitoring method was implemented in a smart home, either a smart home laboratory or a real living home.
- Exclusion
  - No sensor technology was applied;
  - Only wearable/implanted sensors were applied;
  - Sensor data were not used for health monitoring;
  - The work only focused on human–computer interaction;
  - The work presented a design/idea only, but no implementation, test, or evaluation;
  - Research was not on humans;
  - Review/survey/vision papers.

When analyzing the full texts, we focused on extracting three sorts of information, i.e., the types of sensors used in the research and their placement (location), the sensor outputs, including physiological signals/parameters, behavioral and environmental information (data), and the main monitoring functions. Besides, we investigated (i) whether a sensor network was formed and what the communication channel was; (ii) the number of subjects participating in the test/evaluation; (iii) whether the experiment was conducted in a real living environment or a smart home laboratory; and (iv) whether privacy issues were taken into account.

## 3. Results

**Figure 2:**
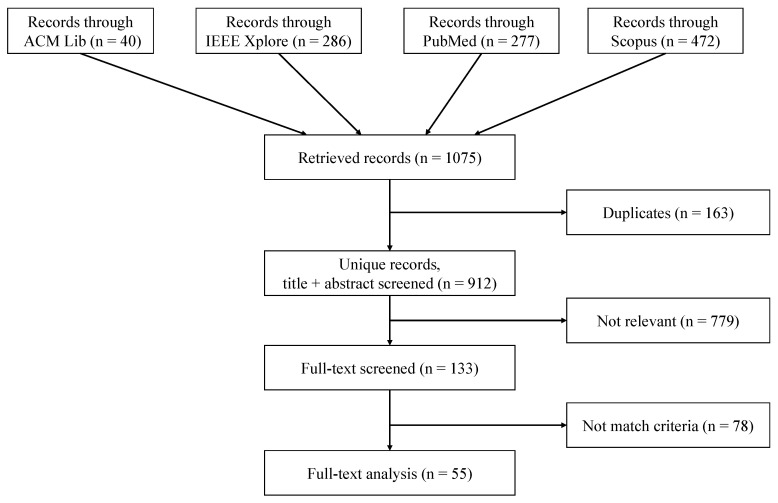
Review flowchart.

The search string on the four databases resulted in 912 records after removing 163 duplicates, of which 133 papers remained after screening titles and abstracts (Figure 2). After reviewing the full texts, we excluded 78 papers as they did not match the criteria, and finally included 55 papers for in-depth text analysis.

Results of the text analysis are given in Table 1 and Table 2. The included papers are sorted by ascending publish year. With the terminology (Figure 1), we extracted the sensors and their deployed locations (represented with the syntax sensor [location]), the acquired data, and the functions of the monitoring system. Additionally, we also examined the type of sensor network, the subject information, and experiment settings, if the information is available.

### 3.1. Sensors and Locations

**Figure 3:**
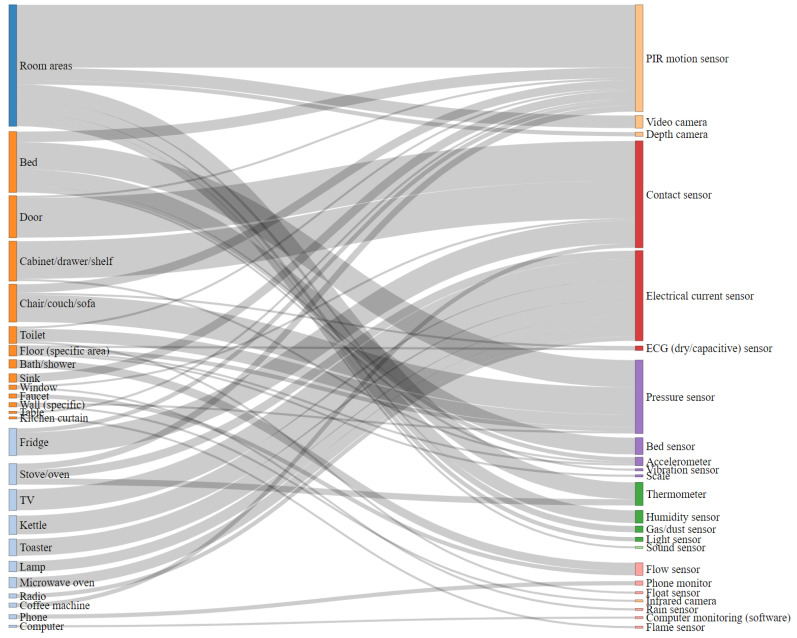
Connections between sensors and their locations. A wider connection indicates more included papers supporting the connection in this review. The terms in the same category are illustrated in the same color.

**Figure 4:**
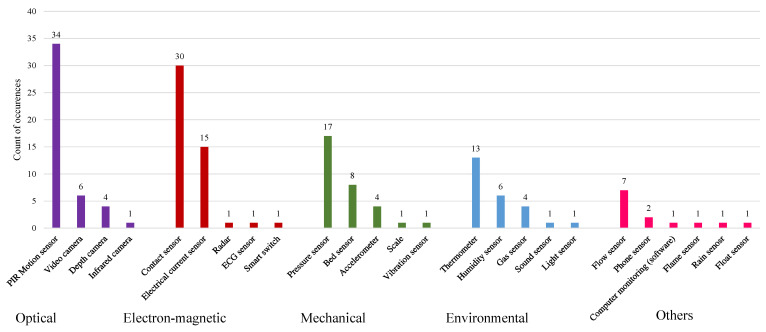
Distribution of sensor occurrences.

A Sankey diagram [79] provides an overview of the connections between sensors and their locations (Figure 3). We found a total of 25 types of sensors in the 55 included papers. Passive infrared (PIR) motion sensors, contact sensors, pressure sensors, and electrical current sensors were the most popularly reported sensing devices, which can be found in 34, 30, 17 and 15 papers, respectively (Figure 4). Many studies used these sensors to monitor human behavior, such as presence and time spent on activities (time on) [26,37,50,60,62,64,76,77,78]. For instance, PIR motion sensors frequently monitored presence in certain room areas. In some cases, however, the motion sensors also detected presence at some specific locations, e.g., stove/oven, toilet, sink, and table/desk [27,60,64,78]. In line with their working principle, contact sensors mostly detected the operations on facilities with doors, such as fridges, shelves, cabinets, and windows [24,51]. Pressure sensors were usually attached to the objects that can undergo pressure due to human’s standing, sitting, or lying. Furniture such as a chair/couch/sofa and bed are the common locations for this kind of sensor [26,41,43,71,76]. As an electrical current sensor detects electric current, we could monitor any electric appliance in theory. In the included work, stoves/ovens and water kettles, which can indicate nutrition activities, were of particular interest [33,47,62,73]. Besides PIR motion sensors, video cameras and air-relevant sensors also monitored room areas [34,35,48,56]. Some research designed customized sensors to monitor the operations of some specific objects. For instance, water flow sensors monitored the use of water supply facilities, a phone integrated with a monitor component monitored its usage, and similarly, monitoring software recorded the computer users’ activities [25,45,50,51]. In addition, we also observed that wearable sensors were also in use in combination with ambient sensors in 10 included papers. The wearable devices are frequently embedded with accelerometers [69,75,76] and radio-frequency identification (RFID) tags [44].

### 3.2. Data

We obtained 20 data types through the introduced combinations of sensors and locations. In line with the sensor occurrence distribution (Figure 4), behavioral data were the most common outputs, with the presence being the most frequent data type (n=39). As the sensors that can detect human–object contact shall deliver presence information, the setups for detecting presence typically included optical sensors, such as PIR motion sensors; contact sensors; and mechanical sensors, such as pressure sensors, accelerometers, and bed sensors. Based on presence data, the time spent on activities was also frequently derived in behavior monitoring (n=18). Besides, a customized placement design of PIR motion sensors estimated the specific metric, the walking speed [27]. Surprisingly, we found rare research on unobtrusively collecting physiological data collection (n=5). Dry or capacitive electrodes appeared to be an alternative solution for capturing ECG signals, from which the heart rate was derived [53]. The body weight (body mass) can be easily measured through a pressure sensor [26]. Besides, research has also derived the blood pressure from the ballistocardiograph (BCG) signal through a pressure sensor [53]. A bed sensor, as an integrated sensor system, successfully delivered heart rate and respiration rate [52]. In room areas, environmental sensors and microphones measured environmental parameters such as gas concentration and sound level, which were used for in-door positioning [48,56].

### 3.3. Monitoring Functions

**Figure 5:**
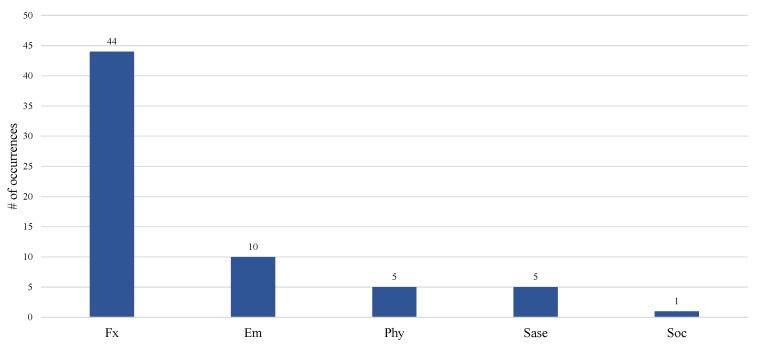
Distribution of smart home functions.

Most included work focused on functional monitoring (n=44). Only a few covered emergency monitoring (n=10), physiological monitoring (n=5), safety and security monitoring (n=5), and social interaction monitoring (n=1) (Figure 5). Intuitively, functional monitoring needed behavioral data, including presence, time spent on activities, walking speed, gait parameters, and time spent out of home. Emergency monitoring relied on video/images, presence detection, and abnormal detection of harmful gas concentrations [29,34,35,61]. Safety and security monitoring used environmental data and presence at the entry door [38,43,48,70]. Only one paper covers the social interaction, in which the phone and computer usage was monitored as the indicator [50].

### 3.4. Sensor Network

**Figure 6:**
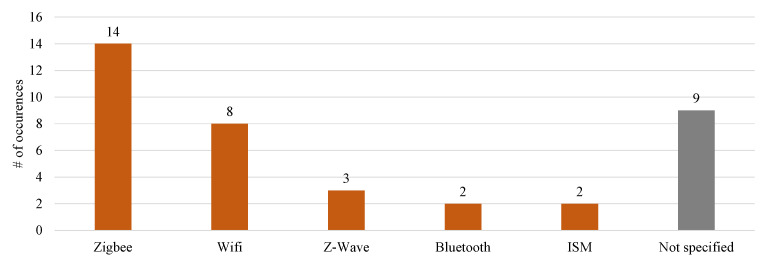
Distribution of wireless sensor networks.

As multiple sensors were used, most works set up a sensor network (n=47) for data transmission, for which 37 papers applied wireless communication. The presented wireless communication protocols include Zigbee, WiFi, Z-Wave, Bluetooth, and ISM bands, found in 14, 8, 3, 2, and 2 papers, respectively (Figure 6).

### 3.5. Subjects and Experimental Settings

**Figure 7:**
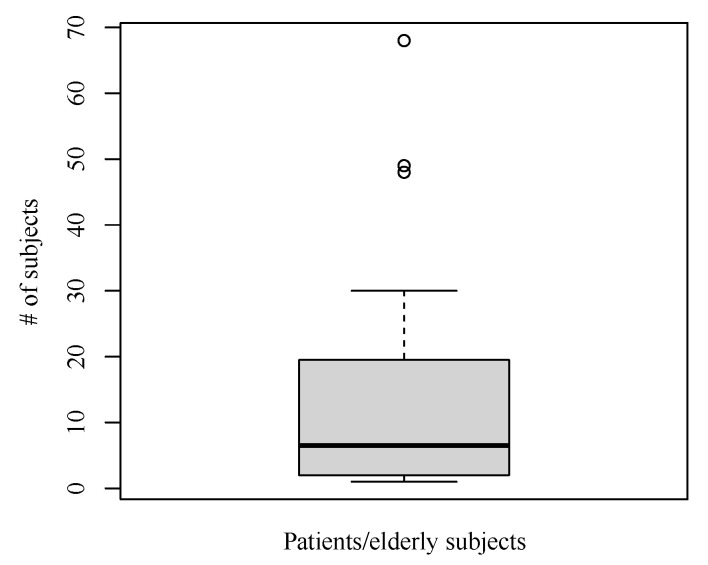
Distribution of number of patients and elderly adults.

The majority of studies (n=36) recruited patients or elderly, whereas some only tested their system with healthy volunteers, e.g., students or developers themselves. Among the research with patients or elderly adults, the number of subjects ranged from one to 265 [27]. The average number was 13.64 without considering the outlier 265 (Figure 7). The 25% and 75% quantiles were 2.0 and 19.25, respectively. Most of the research with patient or elderly adults deployed the monitoring systems in real homes. Only three were used in smart home laboratories. Besides general aging issues, the diseases involved in the included papers were dementia, heart disease, and stroke [26,52,53,60,73,77].

Overall, most research adopted real home settings (n=44), either with real patients/elderly adults or healthy volunteers. The monitoring duration in real homes was much longer than in smart home laboratories. In real home settings, some papers (n=19) reported that the monitoring duration was between one month and one year, while several lasted longer than one year (n=8). In contrast, for the research conducted in smart home laboratories, only one exceeded one month, and the rest were up to one week.

### 3.6. Privacy Issues

Only less than half (n=21) of the included papers mentioned privacy issues. Cameras were referred to in several papers as invasive monitoring [37,38,40,54]. Due to the intruding of privacy, some actions were taken regarding sensor selection and sensor placement. Some cases did not take cameras or vision sensors into account [30,31,33,38]. Due to privacy, the toilet could be sensor-free [72]. For data security, some research has adopted different solutions: raw data were not transmitted [35,76], a sensor network was isolated [42], data transmission was encrypted [50], and data access was authorized [40,43].

### 3.7. Data Sources

Despite most research collecting data using their own monitoring systems, a few (n=8) applied their algorithms on existing datasets [24,30,36,41,55,64,68,74]. The reused external datasets are (i) MavHome and CASAS dataset [80,81] (n=5), (ii) the MIT dataset [82] (n=1), (iii) ORCATECH dataset [27] (n=1), and (iv) TigerPlace dataset [83] (n=1).

## 4. Discussion

To sample relevant literature, in this work, we performed a search with a limited search space on four databases, namely, ACM Digital Lib, IEEE Xplore, PubMed, and Scopus, for acquiring the work in the field of in-home health monitoring published in the past decade. This search strategy might not be able to provide an exhaustive and comprehensive literature coverage; however, we assume that the sampled literature adequately reflects the current state of the research on the topic of unobtrusive health monitoring in smart homes. The high sensitivity of this retrieval strategy is indicated by the fact that approximately 6% of the initially returned records were included for in-depth text analysis (55/912 = 6.03%). We developed a structured terminology for unobtrusive in-home health monitoring (Figure 1). Based on the terminology, we reviewed the included literature in a structured manner. Returning to the initial questions proposed at the beginning (Section 1), we answer them as follows:

- What types of sensors can be used for unobtrusive in-home health data acquisition? To unobtrusively monitor behavior, we can use PIR motion sensors, contact sensors, pressure sensors, and electrical current sensors. Bed sensor systems (respiration rate and heart rate) and dry or capacitive electrodes (ECG, heart rate) are the alternatives to unobtrusively deliver physiological parameters. Apart from that, force-based sensors cal also acquire physiological parameters such as heart rate or blood pressure that can be derived from BCG. Gas sensors, humidity sensors, thermometers, and microphones can be easily unobtrusively deployed for environment monitoring.
- Where should the sensors be placed? Electric appliances and static facilities are the positions for unobtrusively placing the sensors. When monitoring room areas, PIR motion sensors or other optical sensors, and environmental sensors should be placed at appropriate locations according to the sensor fact data. To monitor the presence at a specific location, (i) the facilities with doors are the locations best used to attach contact sensors; (ii) the power supply (e.g., plugin) of a monitored appliance must be able to sustain an electrical current sensor; (iii) a pressure sensor must be beneath the area where force is applied when standing, sitting, walking, or lying; (iv) a position from which a PIR motion sensor can point at the monitored location must be determined to place the sensor according to its fact data. To monitor certain physiological parameters such as heart rate or respiration rate, the positions (e.g., chair and bed) where a person maintains stable contact with the body are appropriate for attaching dry or capacitive electrodes. In this case, acceptable textile layers are the precondition. These locations also qualify for BCG sensors.
- What data can be monitored in the smart home? Behavioral data (presence, time spent on activities, activity level) can be easily acquired through the ambient, sensors such as motion, contact, and pressure sensors. Human functional data such as gait velocity and step time can be derived from depth videos. By customizing the placement of PIR motion sensor, the walking speed can also be estimated. Even though physiological parameters cannot be unobtrusively obtained as easily as behavioral data, heart rate can be derived from BCG (pressure sensor, bed sensor) or ECG (dry or capacitive ECG electrodes), respiration rate can be delivered by bed sensors, and body temperature from an infrared thermometer. Air-relevant and sound sensors can offer environmental data, such as gas concentrations, humidity, and sound level.
- How can the obtained data support the monitoring functions? All five functional categories can be supported by the data from unobtrusive in-home health monitoring systems. Functional monitoring is the easiest one to implement as the variety and the readiness of sensor technology for behavioral monitoring. Emergency monitoring can be built up on behavioral data (e.g., in-door positioning, time-spent on activities, and activity level) and environmental data (e.g., gas level). The physiological data (BCG or ECG) collected from the objects with stable contact (e.g., bed and chair) can deliver heart rate and respiratory rate, leading to partly physiological monitoring. Besides contributing to functional and emergency monitoring, environmental data and behavioral data detect safety and security abnormalities. Social interaction monitoring relies mainly on monitoring social interaction tools, such as phones and computers.

### 4.1. Implications of In-Home Health Monitoring Terminology

The developed terminology of unobtrusive in-home health monitoring (Figure 1) was applied to the structured text analysis in the current work. We expect to generalize the text analysis mechanism to future relevant work. It may serve as a basis for developing a guideline for sensor deployment in this context. Furthermore, it may assist in designing a monitoring system and analyzing it comprehensively. As technology is continuously being developed in sensors and computing, the terminology remains open, and new entries can be added to any dimension.

### 4.2. The Demand for Customized and Hybrid Sensor Technology

Even though many efforts have explored different sensors for in-home health monitoring, many sensors in use, however, are not originally designed for health monitoring. For example, PIR motion sensors are designed initially for presence detection [84], and basic home automation functions like switching on/off lights according to the presence. They may have either blind spots or overlaps in the sensing areas when used for behavioral monitoring, downgrading their value for health monitoring. The sensor systems dedicated designed for unobtrusive health monitoring such as the EarlySense bed sensor [52] are still in demand. Mature products are very likely to improve user experience and enable reliable outputs. Smart building developments will enrich the variety of such simple and non-health-focused sensors, which can yet be used to extract health-related data by sensor fusion. In addition to the static objects that were on the focus of this work, ambient sensors may also be embedded in the mobile objects on which some special groups continuously rely in daily life, for instance, the wheelchairs and crutches for disabled individuals [85,86]. Hence, the monitoring might be extended to other private spaces, such as a smart vehicle [7], and beyond private spaces.

### 4.3. Wearables as Complements to Ambient Sensors

We focused on ambient (non-wearable) sensors that can collect data in an unnoticeable way in this work. Some cases, however, involved wearable sensors as well. For instance, activity monitoring task frequently adopts wearable accelerometers, which can deliver more precise results (e.g., activity level) than ambient sensors [69,75,76]. RFID tag may address the issue of distinguishing multiple individuals under monitoring [44], especially when cameras are absent. Although the ambient sensors have certain advantages, they are also more sensitive to external noise [87]. Given the limited scale of research on physiological monitoring, wearable sensors are advantaging in doing the task. For instance, the commercially available smart watches, smart wristbands (e.g., Jawbone and Fitbit), and smart rings (e.g., Aura) integrated with a photoplethysmogram (PPG) sensor can deliver heart rate, heart rate variability (HRV), respiratory rate, and body temperature [18,88]. Therefore, in current stage, a combination of both types of sensors would be logical if the effort to use wearable sensors can be kept at a minimal level.

### 4.4. The Demand for Appropriate Data Interpretation and Medical Value

In-home health monitoring involves a variety of sensors (Section 3.1) and delivers rich data (Section 3.2). Our results reveal that the majority of research focused on functional monitoring of the elderly or the patients with mental health problems such as Alzheimer’s disease (AD), for whom behavioral changes reflect health status (Section 3.3). Only a few (n=3) papers involved patients with heart disease for whom monitoring physiological parameters (e.g., heart rate) is necessary. The phenomenon might be caused by the difficulty of unobtrusively monitoring physiological parameters, as shown in the results (Section 3.2). Physiological, behavioral, and environmental data are the directly obtainable data. Psychological data or information can be derived from physiological and behavioral data and be affected by environmental factors. Finding a common approach to interpret different sensor data is unfeasible. However, linking the categorized sensor data to the scales of clinical instruments might be possible.

So far, studies with a large sample size and long-term monitoring were rarely conducted (Section 3.5). Convincing evidence for the impact of in-home health monitoring on clinical outcomes is still lacking. However, we identified, despite the limited sample size, recent studies showing promising clues in the direction of evidence, particularly in supporting cognitive impairment. Lussier et al. found that the measures based on sensor-based observations (motion, contact, and electric sensors) associated with daily functional performance of older adults and concluded that sensor technology hold potential in detecting MCI [73]. By conducting an observational study, Lazarou et al. concluded that unobtrusive health monitoring has positive impact on guiding intervention to the caring of patients with cognitive impairment [76]. As some disease progression can be slow, randomized clinical trials aiming to demonstrate improved patient health outcomes shall be conducted for many years to reach statistical significance [89]. We encourage evidence-oriented research to offer meaningful medical values of unobtrusive in-home health monitoring [90].

### 4.5. Wireless vs. Wired Sensor Networks

Wireless communication is a dominant approach to form a sensor network (Section 3.4). Many advantages make wireless communication superior, such as simplified installation, flexibility to the building structure, low costs, and good support for IoT sensors. While all of these advantages popularize wireless sensors, short-term projects (like pilot studies) do not allow permanent installation in scales of building usage duration (typically 30 years). Therefore, wired sensors come into play. They allow an efficient and unobtrusive long-term integration of a broad range of sensors and actuators with the reliability needed by health-related applications and the scalability required for large and long-term trials [91]. A hybrid model would bring both into full play. Secondarily using existing sensors, installed by housing companies or homeowners for comfort or safety and security reasons, to collect in-home health data [92], and then fusing with wearable sensor data, could be a realistic model.

### 4.6. The Demand for Open Data Sources

We also observed that some papers were based on publicly accessible datasets. In contrast to the open data availability in public health or bioinformatics, there are still rare datasets of in-home health monitoring shared within the research community. The reason for that is evident: Collecting sensor data in the real life of patients or the elderly is expensive, particularly, from the perspective of time. The secondary usage of existing data shall be encouraged to promote the advancement, above all, in developing machine learning methods. Open data policy in levels could be a feasible approach. For example, some datasets in MavHome/CASAS are free to download, whereas some can only be accessed by proposing an application [93]. To ensure the usability, open data sources must provide well-defined and de-identified metadata as well.

### 4.7. On Data Processing

This work focused on giving an overview of sensor technology and measured health-relevant data in state-of-the-art unobtrusive health monitoring applications. However, we did not focus on the processing techniques when extracting the health information from the measured data. The reason for that lies within the fact the majority of works focuses on sensor data collection and on data processing only to a lesser extent. This is underlined by the fact that 20% of works did not report on the data processing technique. Therefore, there is no clear picture of the used techniques for data processing. In some cases, straightforward techniques (thresholds, frequency distributions, distance functions) or statistical measures (nearest neighbor, linear regression) are applied. Rare cases used machine learning techniques, such as support vector machines and recurrent neural networks. Their dependence on training data, which are costly and time-consuming to acquire in the unobtrusive health monitoring, could explain the fact.

To our knowledge, physiological data collection in unobtrusive in-home monitoring is usually over a long-term. Meanwhile, real life introduces noise sources (e.g., movements), resulting in low signal quality. Data quality assessment methods are required. Therefore, collecting high-quality, large-scale training data in an open format that allows mapping this data to other projects effortlessly is an avenue for future work.

### 4.8. On Privacy Issues

Last but not least, privacy issues are unavoidable in implementing health monitoring in a private space. Sensors that can intrude privacy shall be avoided. In the papers in this work, a video camera was not used in any research that has done monitoring for longer than a month in real homes. However, a depth camera could be an alternative sensor to balance privacy protection and the richness of delivered information. Only less than half of the included papers considered privacy issues, which may also explain the general few numbers of subjects and short duration of monitoring (Section 3.5). In a design stage, the well-known Fair Information Practice Principles may serve as a guideline or reference for protecting privacy, including seven principles on openness and transparency, individual participation, collection limitation, data quality, use limitation, reasonable security, and accountability [94,95]. Additionally, the Model for the Ethical Evaluation of Socio-Technical Arrangements (MEESTAR) [96] offers a structured way to identify ethically problematic effects.

## 5. Final Remarks

- The terminology of unobtrusive in-home health monitoring enables a structured analysis of health monitoring in the smart home environment, and may contribute to guiding sensor deployment in in-home health monitoring, designing a monitoring system, and analyzing it comprehensively.
- Locations in a home environment, categorized into room areas, electric appliances, and static facilities, can unobtrusively hold a diversity of sensors (mechanical, electro-magnetic, optical, etc.).
- While behavioral data can be easily acquired, only limited types of physiological parameters are unobtrusively measurable. Physiological sensor technology needs to be further developed to enable more reliable outputs for an ambient placement.
- A combination use of the sensor data makes the smart home a platform for functional, emergency, physiological, safety and security, and social interaction monitoring.
- Convincing proof of a clear effect of these monitoring functions on some clinical outcome using a large sample size and long-term monitoring is still lacking. Sensor data need to be interpreted with corresponding medical concerns to obtain insights.
- Open data policies in this research field should be encouraged to enrich the available data to develop and evaluate new methods.
- Privacy issues must be guided by frameworks that are convincing for multiple stakeholders for the sake of long-term monitoring in practice.

## Figures and Tables

**Table 1:** Recent research on in-home health monitoring. NA: not available. RH: real home. SHL: smart home laboratory.

Ref.	Year	Sensors & Locations	Data	Functions	Sensor Network	Subject Info	Experiment Setting
[24]	2010	Contact sensor [doors, windows, cabinets, sinks, toilets, and electric/electronic appliances (e.g., ovens & fridge)	Presence (use of objects)	Fx: ADL recognition & monitoring	Yes, wireless, not specified	2, 30-year and 80-year	RH, 14 days
[25]	2010	{Thermometer, humidity sensor} [above cooking area], flow-meter [water flowing], contact sensor [shelves, fridge, drawers], motion sensor [kitchen], camera [kitchen ceiling]	Actions and events during cooking, increased/decreased temperature/humidity, position	Fx: ADL and nutritional habits	NA	1, unkown info	SHL, duration NA
[26]	2011	Infrared camera [wall in living room], scale [floor], pressure sensor [floor, living room]	Body temperature, body weight, presence	Phy: vital sign monitoring for heart disease patients	Yes, not specified	28, 13 with heart disease, and 15 healthy	SHL, < 1 hour
[27]	2011	Motion sensors [room areas, sofa, kitchen area, toilet, bed], contact sensor [(exit/entry) doors, fridge]	Presence, walking speed, out of home (absence)	Fx: Assessment of aging	Yes, wireless, X10	265, elderly persons	RH, average 33 months
[28]	2011	Electrical current sensor [microwave oven, kettle, TV, toaster, bed lamp], pressure sensor[bed]	Time spent on act. (duration of using appliances)	Fx: ADL monitoring for elderly	Yes, Xbee based on Zigbee	1, healthy volunteer	RH, 24 hours
[29]	2011	Motion sensor [room areas], contact sensor [front&back doors]	Presence	Em: Abnormal behavior	Yes, not specified	1, info NA	RH, 1.5 years
[30]	2012	Motion sensor [room areas]	Presence, time on act.	Fx: Anomaly detection of behavioral patterns	Yes, wired, X10	1, healthy volunteer	SHL, 2 months
[31]	2012	Electrical current sensor [toaster, oven, kettle, TV, lamp], pressure sensor [bed, chair, toilet], contact sensor [fridge, cabinet], water flow sensor [bath]	Presence, time on act. (duration of using appliances)	Fx: Wellness assessment	Yes, ZigBee	4, elderly	RH, 6 days
[32]	2012	Electrical current sensor [toaster, oven, kettle, TV, lamp], pressure sensor [bed, chair, toilet], contact sensor [fridge, cabinet], water flow sensor [bath]	Presence, time on act. (duration of using appliances)	Fx: Sleeping activity monitoring, forecasting sleeping tendency	Yes, ZigBee	1, elderly	RH, 8 weeks
[33]	2012	Electrical current sensor [toaster, oven, kettle, TV, lamp], pressure sensor [bed, chair, toilet], contact sensor [fridge, cabinet], water flow sensor [bath]	Presence, time on act. (duration of using appliances)	Fx: assessing performance of basic behaviors	Yes, ZigBee	4, elderly	RH, 6 days
[34]	2012	Video camera [bed room]	Video of ADL	Em: Fall detection	No	NA, healthy volunteers	RH, duration NA
[35]	2012	Video camera [living room]	Video of ADL	Em: Fall detection	No	15, healthy volunteers	RH, duration NA
[36]	2012	Motion sensor [room areas], bed sensor [bed], thermometer [stove]	Activity level in apt. and in bed (bed restlessness), heart rate (low pulse), respiration rate (low breathing)	Phy: Early illness recognition for older adults	Yes, X10	6, elderly	RH, 1 month -– 2 years
[37]	2013	Thermometer [stove], Doppler radar sensors [NA], depth camera (MS Kinect) [NA], motion sensor [room areas], bed sensor (pneumatic strip) [bed]	Activity level in apt. and in bed (bed restlessness), heat rate (low/high pulse), respiration rate, time on act. (stove usage), gait (velocity and step time)	Fx, Phy: Detect changes in health status	Yes, X10	49, 24 discharged, 25 remained	RH, 1 year
[38]	2013	Smart lamp (motion sensor, thermometer, humidity sensor, gas sensor) [room areas], + wearable (smart watch)	Presence, gas leak, temperature, humidity	Safety/SaSe: for down syndrome	Yes, ISM 868MHz	NA	SHL, 2 days
[39]	2013	Electrical current sensor [room heater, kettle, toaster, microwave, TV, and dishwasher], pressure sensor [bed, couch, chair, toilet], contact sensor [fridge, cabinet]	Presence, time on act. (duration of using appliances)	Fx: Predicting the quantitative well-being of an elderly	Yes, ZigBee	1, elderly person	RH, 8 weeks
[40]	2013	Motion sensor [room areas], contact sensor [cupboard], light sensor [storage, door], thermometer [stove, bathroom], electrical current sensor [kettle, toaster, wash machine], water flow sensor [sink in kitchen & bathroom], humidity [bathroom]	Presence, appliance use, temperature, humidity, brightness	Fx: ADL monitoring	Yes, wireless, proprietary protocol	1, elderly	RH, duration NA
[41]	2013	Pressure sensor [bed, toilet, couch, chair], electrical current sensor [TV, heater, kettle, toaster, microwave], contact sensor [cupboard]	Presence, time spent on act. (appliances)	Fx: ADL monitoring	yes, wireless, Xbee (Zigbee-based)	4, elderly	RH, 10 weeks
[42]	2014	(Low-resolution) cameras [room areas]	(low resolution) images, similarity heatmaps	Fx: ADL monitoring	Yes, wired, not specified	1, 80-year, with hearing impairment and walking abnormalities	RH, 14 days
[43]	2014	Bed sensor [bed], pressure sensor [chair cushion], contact sensor [door], + wearable sensor	Presence (bed, chair, entry/exit)	SaSe & Fx: ADL monitoring of patients with AD	Yes, wireless, ZigBee	14, patients with AD	RH, duration NA
[44]	2014	Pressure sensor [carpet in bedroom], contact sensor [door], bed sensor (air pressure sensor) [bed], motion sensor [NA], + wearable (RFID tags)	Presence, out of home, fall	Fx: ADL and emergency monitoring	Yes, Xbee	6, healthy volunteers	SHL, a few mins
[45]	2014	{Contact sensor, accelerometer} [cabinet (pill box)], phone sensor [phone], contact sensor [coffee maker]	Presence (medication taking, coffee making), phone usage	Fx: ADL monitoring	Yes, wireless, not specified	Study-1: 2 older women; study-2: 12 older adults, living alone.	RH, 10 months
[46]	2015	Motion sensor [room areas], contact sensor [doors], bed sensor [bed], + wearable sensors	Activity level (# sensor firings, # of transition between rooms, in-bed movements), time on act. (time spent per location)	Fx: ADL monitoring	Yes, not specified	7, info NA	SHL, 4 days
[47]	2015	Video camera [kitchen], electric current sensor [TV, iron, vacuum, cooking devices, boiler, radio], contact sensor [TV, iron, vacuum, fridge door, drug cabinet, drug box], motion sensor [kitchen, bathroom], bed sensor [bed], + wearable (Jawbone)	Images, sleep interruption, out of home, presence	Fx: ADL monitoring	Yes, wireless	1, 76 years old, female	RH, 3 months
[48]	2016	Motion sensor [room areas], gas sensor (CO, air quality, smoke) [room areas], humidity sensor [room areas], thermometer [room areas], sound sensor [room areas]	Presence, humidity, temperature, gas concentration (CO level), sound level.	SaSe: detect or prevent domestic emergency/abnormal situations	Yes, Ethernet, WiFi, GPRS	7, healthy volunteers	RH, 15 days
[49]	2016	Motion sensors [room areas], + wearable sensors	Presence	Fx: ADL tracking	Yes, Bluetooth	20, healthy volunteer, 20–79 years	RH, duration NA
[50]	2016	Motion sensor [room areas], contact sensor [doors], phone sensor [phone], computer (monitoring software)	Out of home, walking speed, phone usage, time on act. (computer)	Soci: assessing/predicting loneliness	Yes, WiFi, USB cable	16, older adults (>62), living alone	RH, 8 months
[51]	2016	Motion sensor [room areas], contact sensor [fridge, cabinet], pressure sensor [bed, chair], water flow sensor [valve], electric current sensor [TV, radio]	Presence	Fx: Profile nighttime routines, detecting wandering	Yes, Z-wave, WiFi	1, healthy volunteer	RH, 3 months

**Table 2:** Recent research on in-home health monitoring (continued). NA: not available. RH: real home. SHL: smart home laboratory.

Ref.	Year	Sensors & Locations	Data	Functions	Sensor Network	Subject Info	Experiment Setting
[52]	2016	Bed sensor (EarlySense piezoelectric sensor) [bed, under the mattress]	Heart rate, respiration rate (rapid and shallow respiration duration), activity level	Phy: Assessing change of physiological patterns correlate with readmission	Yes, LAN or WiFi	30, patients with systolic left ventricular dysfunction, and those with preserved ejection fraction	RH, 640 nights
[53]	2017	Accelerometer [blanket in bed], pressure sensor [chair, bed, floor tile in bathroom], ECG sensor (capacitive electrodes) [chair at dining table, couch]), ECG sensor (dry electrodes) [floor tile in bathroom], Infrared thermometer [at TV]	ECG, heart rate, respiration rate, weight, body temperature, BCG (at the chair), blood pressure	Phy: perceptions of seniors with heart failure	Yes, wireless, not specified	26, heart failure, >65 years, living alone	SHL, <1 h
[54]	2017	pressure sensor (handrail) [wall of hallway to toilet]	Presence, walking speed	Fx: ADL monitoring	No	1, elderly (88-year)	RH, 14 months
[55]	2017	Motion sensor [room areas, bed], contact sensor [doors, fridge]	Activity level (activity distribution per location)	Fx: correlation between activity distribution and MCI	Yes, X10	68, aged > 70, living independently, some experiencing MCI)	RH, average 3 years
[56]	2017	Thermometer [room areas], gas sensor (CO2) [room areas], humidity sensor [rooms areas]	Temperature, humidity, gas level (CO2), presence	Fx: Residence position	Yes, WiFi	1, volunteers	RH, a few hours
[57]	2017	Motion sensor [room areas], electrical current sensor [stove/oven, kettle, microwave, etc.], accelerometer [bed], contact sensor [doors], water flow sensor (acoustic) [water sink], temperature/humidity sensor [room areas]	Presence, time spent on act., temperature, humidity, out of home	Fx: ADL routine	NA	5, age > 70	RH, 181 days
[58]	2017	Depth camera [room areas]	Depth image, motion trajectory	Fx: ADL monitoring, abnormal detection	NA	4, elderly, cognitive problem, Parkinson’s disease	RH, 40–78 days
[59]	2017	Contact sensor [doors, cupboard, toilet flush tank, garderobe, water faucet], pressure sensor [chair, bed], thermometer [oven], motion sensor [bath], light sensor [room areas], flame sensor [kitchen curtain], rain sensor [kitchen window]	Presence, environmental data (light, rain, flame)	SaSe & Fx: ADL monitoring, forgotten situations	Yes, wireless, Xbee	1, living alone	RH, duration NA
[60]	2018	Motion sensor [room areas, chairs, bed, stove, sink, and fridge]	Time on act. (cooking, eating, relaxing movements, and hygiene act., night toilet, out of home, sleep), gait (walking distance)	Fx: assessing functional health decline	Yes, ZigBee	29, older adults, 13 cognitively healthy, 10 at risk of cognitive difficulties, 6 cognitive difficulties	RH, >2 years
[61]	2018	Depth cameras (MS Kinect) [-], accelerometer (floor tile in kitchen]	Depth images (walking, standing, sitting, falls, position), presence (via accelerometer)	Fx & Em: In-home ADL recognition and tracking, fall detection	Yes, ZigBee	6, volunteers	SHL, duration NA
[62]	2018	Motion sensor [door, sink in kitchen], electric current sensor [kettle, rice cooker, microwave, TV]	Time on act.	Fx & Em: ADL monitoring	Yes, wireless 2.4G ISM Bands	4, elderly	RH, 7 weeks
[63]	2018	Motion sensor [room areas, bed], contact sensor [doors, drawer, wardrobe], electric current sensor [lamps, TV, coffee machine]	Presence	Fx: ADL prediction to support older adults	Yes, Z-Wave, xComfort	10, elderly patients	RH, up to 17 weeks
[64]	2018	Motion sensor [room areas, chairs, bed, stove, sink, and fridge]	Time on act. (cooking, eating, relaxing movements, and personal hygiene activities, night toilet, sleep), out of home, walking distance	Fx: Symptom prediction of AD patients	Yes, ZigBee	29, older adults, 13 cognitively healthy, 10 at risk of cognitive difficulties, 6 cognitive difficulties	RH, >2 years
[65]	2018	Motion sensor [room areas], vibration sensor [bed], thermometer [bed room]	Presence, temperature	Fx: Detecting early symptoms of MCI	NA	50, info NA	RH, 6 months
[66]	2018	Contact sensor [doors, medicine cabinet], motion sensor [bed room], pressure sensor [couch], photo sensor [TV], + wearable (BodyMedia)	Presence, time spent on act.	Fx: ADL monitoring, adhere to self-management regimens	Yes, wireless, not specified	2, 82-year old, male; 60-year-old female; both with type II diabetes	RH, 1–2 months
[67]	2018	Motion sensor [room areas], contact sensor [door, drawer, cabinet], smart switch [electrical appliances]	Presence	Fx: ADL routine	Yes, wireless, not specified	7, average 82-year	RH, 8 weeks
[68]	2018	Motion sensor [room areas], contact sensor [doors], thermometer [NA], light sensor [NA]	Presence, time spent on act., activity level	Functional monitoring: ADL monitoring, health prediction	NA	10, elderly (80–91), five with MCI	RH, a few months
[69]	2019	Motion sensor [room areas], pressure sensor [chair, bed], contact sensor [door], + wearable sensors	Presence (kitchen, bathroom, hall), time on act. (bed, chair), out of home	Fx: Tracking activity and sleep patterns	Yes, wired, phone lines	10, female, living alone, average 86.5	RH, 3 months
[70]	2019	{Thermometer / air quality (gas) sensor} [room areas], conductive cushion sensors [wheelchair], camera [bed room]	Temperature, humidity, gas concentration (VoC, fine dust, pollution level), presence, images	Em& SaSe: Unsafe situation detection	Yes, WiFi	1, healthy volunteer	SHL, duration NA
[71]	2019	Motion sensor [room areas], pressure sensor [slipper, sofa, bed, toilet, chair]	Presence, time on atc.	Fx & Em: ADL monitoring, abnormal activities detection	Yes, WiFi, Bluetooth	1, healthy volunteer	RH, 48 hours
[72]	2019	Depth camera (MS Kinect) [room areas]	(Depth) images, presence	Fx and Em: out of home	Yes, wired, not specified	# NA, healthy volunteers	SHL, duration NA
[73]	2019	Motion sensor [room areas], electric current sensor [coffee maker, toaster], contact sensor [drawer, fridge, cupboards]	Time spent on act. (some areas usage time)	Fx: Measuring the performance of specific tasks	Yes, Z-Wave	48, 26 cognitive healthy, 22 MCI	SHL, ca. 4 hours
[74]	2019	(1) Motion sensor [room areas, chairs and bed, stove, sink and fridge]; (2) contact sensor [door, cupboards], pressure sensor [couch, bed, drawer], motion sensor [room areas], water flow sensor [toilet]	Presence (use of obj.)	Fx: ADL monitoring, activity routines	(1) Yes, Zigbee (2) Yes, RFM wireless network	1, healthy adult	RH, 1 month
[75]	2019	Motion sensor [room areas], contact sensor [doors], + wearable sensors	Presence	Fx: ADL monitoring patients with Parkinson’s disease	Yes, Zigbee	4, 2 male, 2 female, age 65–70, PD disease duration 10 - 14 years	RH, 4 weeks
[76]	2019	Electric current senor [electronic appliances], contact sensor [drug box, water can], motion sensor [room areas], bed sensor [bed], depth camera [NA], + wearable (Jawbone)	Images, depth data, gestures, activities, devices in use, presence, time spent on atc. (sleep), activity level (steps)	Fx: ADL monitoring of patients with cognitive impairment	Yes, wireless, NA	18, 12 MCI, 6 AD, >70 years	RH, 4–12 months
[77]	2019	Contact sensor [door], motion sensor [room areas], electric current sensor [NA]	Behavior routine	Fx: ADL monitoring, routine monitoring	Yes, wireless, NA	19, stroke survivors, 9 female and 10 male, mean age 71 (SD 11)	RH, 8 weeks
[78]	2019	Motion sensor [bathroom, bed, dining table, desk], thermometer [hallway]	Presence (sleep, meal, TV)	Fx: ADL monitoring	Yes, Wifi	1, female, 68 years	RH, 5 days

## Data Availability

Not applicable.

## Abbreviations

The following abbreviations are used in this manuscript:

AAL	Ambient Assisted Living
AD	Alzheimer’s Disease
ADLs	activities of daily living
BADLs	basic ADLs
BCG	ballistocardiograph
CASAS	Center for Advanced Studies in Adaptive Systems
CO	carbon monoxide
COPD	chronic obstructive pulmonary disease
CO2	carbon dioxide
ECG	Electrocardiography
Em	Emergency detection
GPRS	General Packet Radio Service
HRV	heart rate variability
IADLs	instrumental ADLs
IoT	Internet of Things
ISAAC	Intelligent Systems for Detection of Aging Changes
ISM	industrial, scientific and medical
LAN	local area network
MCI	Mild Cognitive Impairment
MEESTAR	Model for the Ethical Evaluation of Socio-Technical Arrangements
MIT	Massachusetts Institute of Technology
NA	not available
ORCATECH	Oregon Center for Aging & Technology
Phy	physiological monitoring
PIR	passive infrared
PPG	photoplethysmogram
RH	real home
RFID	Radio-frequency identification
SaSe	safety and security detection
SHL	smart home laboratory
SoC	social interaction monitoring
TV	television

## Appendix A. Search String

### Appendix A.1. ACM Digital Lib

(“in-home monitoring OR home monitoring” OR “home-based monitoring” OR “unobtrusive monitoring” OR “continuous assessment” OR “smart home” OR “smart homes” OR “AAL” OR “ambient assisted living” OR “assistive living” OR “aging in place” OR “intelligent monitoring”)

AND

(“patient” OR “patients” OR “disease” OR “diseases” OR “illness” OR “disabled” OR “geriatric” OR “aging” OR “elderly” OR “senior” OR “seniors” OR “older adults” OR “old adults” OR “people with”)

### Appendix A.2. IEEE Xplore

(“Document Title”:in-home monitoring OR “Document Title”:home monitoring OR “Document Title”:home-based monitoring OR “Document Title”:unobtrusive monitoring OR “Document Title”:continuous assessment OR “Document Title”:smart home OR “Document Title”:smart homes OR “Document Title”:AAL OR “Document Title”:ambient assisted living OR “Document Title”:assistive living OR “Document Title”:aging in place OR “Document Title”:intelligent monitoring)

AND

(“Document Title”:patient OR “Document Title”:patients OR “Document Title”:disease OR “Document Title”:diseases OR “Document Title”:illness OR “Document Title”:disabled OR “Document Title”:geriatric OR “Document Title”:aging OR “Document Title”:elderly OR “Document Title”:senior OR “Document Title”:seniors OR “Document Title”:older adults OR “Document Title”:old adults OR “Document Title”:people with)

### Appendix A.3. PubMed

(in-home monitoring[Title] OR home monitoring[Title] OR home-based monitoring[Title] OR unobtrusive monitoring[Title] OR continuous assessment[Title] OR smart home[Title] OR smart homes[Title] OR AAL[Title] OR ambient assisted living[Title] OR assistive living[Title] OR aging in place[Title] OR intelligent monitoring[Title])

AND

(patient[Title] OR patients[Title] OR disease[Title] OR diseases[Title] OR illness[Title] OR disabled[Title] OR geriatric[Title] OR aging[Title] OR elderly[Title] OR senior[Title] OR seniors[Title] OR older adults[Title] OR old adults[Title] OR people with[Title])

### Appendix A.4. Scopus

TITLE ( ( “in-home monitoring” OR “home monitoring” OR “home-based monitoring” OR “unobtrusive monitoring” OR “continuous assessment” OR “smart home” OR “smart homes” OR “AAL” OR “ambient assisted living” OR “assistive living” OR “aging in place” OR “intelligent monitoring” )

AND

( “patient” OR “patients” OR “disease” OR “diseases” OR “illness” OR “disabled” OR “geriatric” OR “aging” OR “elderly” OR “senior” OR “seniors” OR “older adults” OR “old adults” OR “people with” ) )

AND

( LIMIT-TO ( PUBSTAGE , “final” ) ) AND ( LIMIT-TO ( SUBJAREA , “COMP” ) OR LIMIT-TO ( SUBJAREA , “MEDI” ) OR LIMIT-TO ( SUBJAREA , “SOCI” ) OR LIMIT-TO ( SUBJAREA , “NURS” ) OR LIMIT-TO ( SUBJAREA , “HEAL” ) OR LIMIT-TO ( SUBJAREA , “PSYC” ) OR EXCLUDE ( SUBJAREA , “MATH” ) OR EXCLUDE ( SUBJAREA , “BIOC” ) OR EXCLUDE ( SUBJAREA , “ARTS” ) OR EXCLUDE ( SUBJAREA , “ENER” ) OR EXCLUDE ( SUBJAREA , “BUSI” ) OR EXCLUDE ( SUBJAREA , “CENG” ) OR EXCLUDE ( SUBJAREA , “AGRI” ) OR EXCLUDE ( SUBJAREA , “ECON” ) OR EXCLUDE ( SUBJAREA , “EART” ) OR EXCLUDE ( SUBJAREA , “IMMU” ) ) AND ( EXCLUDE ( DOCTYPE , “re” ) ) AND ( LIMIT-TO ( PUBYEAR , 2020 ) OR LIMIT-TO ( PUBYEAR , 2019 ) OR LIMIT-TO ( PUBYEAR , 2018 ) OR LIMIT-TO ( PUBYEAR , 2017 ) OR LIMIT-TO ( PUBYEAR , 2016 ) OR LIMIT-TO ( PUBYEAR , 2015 ) OR LIMIT-TO ( PUBYEAR , 2014 ) OR LIMIT-TO ( PUBYEAR , 2013 ) OR LIMIT-TO ( PUBYEAR , 2012 ) OR LIMIT-TO ( PUBYEAR , 2011 ) OR LIMIT-TO ( PUBYEAR , 2010 ) ) AND ( LIMIT-TO ( LANGUAGE , “English” ) ) AND ( EXCLUDE ( SRCTYPE , “b” ) ) AND ( EXCLUDE ( DOCTYPE , “ch” ) ) AND ( EXCLUDE ( SRCTYPE , “k” ))
